# Proteomic analysis of human follicular fluid associated with successful in vitro fertilization

**DOI:** 10.1186/s12958-017-0277-y

**Published:** 2017-07-27

**Authors:** Xiaofang Shen, Xin Liu, Peng Zhu, Yuhua Zhang, Jiahui Wang, Yanwei Wang, Wenting Wang, Juan Liu, Ning Li, Fujun Liu

**Affiliations:** 1 Central Laboratory, The Affiliated Yantai Yuhuangding Hospital of Qingdao University, Yantai, 264000 Shandong People’s Republic of China; 2 Reproductive center, Beijing BaoDao obstetrics and gynecology hospital, Beijing, 100000 People’s Republic of China; 3 Reproductive Medicine Centre, Wei fang people’s hospital, Weifang, Shandong 261000 People’s Republic of China

**Keywords:** Human follicular fluid, *in vitro* fertilization, LC-MALDI TOF/TOF MS, Folliculogenesis, Bioinformatics

## Abstract

**Background:**

Human follicular fluid (HFF) provides a key environment for follicle development and oocyte maturation, and contributes to oocyte quality and in vitro fertilization (IVF) outcome.

**Methods:**

To better understand folliculogenesis in the ovary, a proteomic strategy based on dual reverse phase high performance liquid chromatography (RP-HPLC) coupled to matrix-assisted laser desorption/ionization time-of-flight tandem mass spectrometry (LC-MALDI TOF/TOF MS) was used to investigate the protein profile of HFF from women undergoing successful IVF.

**Results:**

A total of 219 unique high-confidence (False Discovery Rate (FDR) < 0.01) HFF proteins were identified by searching the reviewed Swiss-Prot human database (20,183 sequences), and MS data were further verified by western blot. PANTHER showed HFF proteins were involved in complement and coagulation cascade, growth factor and hormone, immunity, and transportation, KEGG indicated their pathway, and STRING demonstrated their interaction networks. In comparison, 32% and 50% of proteins have not been reported in previous human follicular fluid and plasma.

**Conclusions:**

Our HFF proteome research provided a new complementary high-confidence dataset of folliculogenesis and oocyte maturation environment. Those proteins associated with innate immunity, complement cascade, blood coagulation, and angiogenesis might serve as the biomarkers of female infertility and IVF outcome, and their pathways facilitated a complete exhibition of reproductive process.

**Electronic supplementary material:**

The online version of this article (doi:10.1186/s12958-017-0277-y) contains supplementary material, which is available to authorized users.

## Background

In vitro fertilization (IVF) coupled with embryo transfer into uterus has been applied as treatment for infertility several decades. IVF was initially used to assist the reproduction of sub-fertile women caused by tubal factors [1]. With the improvement of IVF techniques, IVF is now a routine treatment for many reproductive diseases. However, the success rate of pregnancy is still a problem in clinical IVF practice, which is only about 50% even if the embryos with normal morphology were used for transfer [2]. In order to select embryos with the best potential good for IVF outcome, morphological assessments of blastocyst and blastocoels have been adopted, but it was still difficult to predict the quality of embryos [3]. Therefore, it was necessary to develop new strategies for embryo quality evaluation. Epidemiologic investigations showed that many intrinsic and extrinsic factors contributed to the quality of embryo. Because oocyte quality directly influences embryo development, HFF (microenvironment of oocyte maturation) became a main factor contributing to the success of IVF treatment [4].

Small antral follicles respond to ovarian stimulation by increasing in size due to rapid accumulation of follicular fluid, as well as granulosa cell divisions, which necessitate follicular basal lamina expansion. The components of HFF had several origins: secretions from granulosa cells, thecal cells, occytes, and blood plasma composition transferred through the thecal capillaries [5]. The major components of HFF were proteins [6], steroid hormones [7], and metabolites [8]. HFF provided a special milieu to facilitate the communications between occyte and follicular cells, the development of follicle and the maturation of occytes. The alteration of HFF proteins reflected disorders of main secretary function of granulosa cells and thecae, and the damage of blood follicular barrier, which was associated with abnormal folliculogenesis [9] and a diminished reproductive potential [10]. In IVF treatment, HFF was easily accessible during the aspiration of oocytes from follicle, and was an ideal source for noninvasive screening of biomakers for oocyte maturation, fertilization success, IVF outcome, pregnancy, and ovarian diseases.

In the postgenomic era, proteomic techniques have been widely used in the field of reproductive medicine. HFF proteome has become a hotspot for research, which not only contributed to discovering proteins related to IVF outcomes, but also improved our comprehensive understanding of physiological process during follicle development and oocyte maturation [11]. Li and co-workers used surface-enhanced laser desorption/ionization-time of flight-mass spectrometry (SELDI-TOF-MS) combined with weak cation-exchange protein chip (WCX-2) to search for differentially expressed HFF proteins from mature and antral follicles [12]. Two-dimensional gel electrophoresis (2D–GE) followed by matrix-assisted laser desorption/ionization time-of-flight mass spectrometry (MALDI-TOF MS) was also used to identify 8 differentially expressed HFF proteins related to immune and inflammatory responses from controlled ovarian hyperstimulation (COH) and natural ovulatory cycles [13]. Ambekar and co-workers carried out SDS-PAGE, OFFGEL and SCX-based separation followed by LC-MS/MS analysis to characterize 480 HFF proteins for a better understanding of folliculogenesis physiology [14]. Chen and co-workers explored the HFF biomarkers between successfully fertilized oocytes and unfertilized mature oocytes through nano-scale liquid chromatography coupled to tandem mass spectrometry (nano LC-MS/MS), and found 53 peptides to be potential candidates [15]. Although proteomic researches on HFF deepened our understanding of reproductive process and provided candidates related to oocyte quality, follicle development, IVF outcome and ovarian disorders, it was still essential to fully delineate the HFF networks and pathways involved in the physiology of reproduction and pathophysiology of infertility.

In the present study, we carried out an in-depth proteomic analysis of HFF from women undergoing successful IVF based on dual RP-HPLC coupled to MALDI TOF/TOF MS. The results profiled candidate biomarkers for the prediction of oocyte maturation, fertilization, and pregnancy and provided a new complement for HFF dataset, which will improve the understanding of biological processes and complicated pathways and interaction networks in HFF.

## Methods

### Patients enrollment and sample preparation

The HFF samples were collected from 10 women who underwent IVF treatment and achieved pregnancy. The selected patients met the following criteria: infertility not caused by tubal factor; aged less than 38 years; serum FSH values <12 mIU/mL; undergoing their first fresh egg retrieval cycle; ovulation stimulated with the long protocol. The patients were also without chromosomal abnormalities, polycystic ovary syndrome (PCOS), endometriosis and or endocrine disease. Cause of infertility was simple male factor. The body mass index (BMI) of patients met the normal criteria proposed by WGOC (18.5 ≤ BMI ≤ 23.9 kg/m^2^) [16–18]. Ovarian stimulation and oocyte retrieval were performed as previously described [19]. Briefly, when more than two follicles exceeded 18 mm in diameter, 10,000 IU of HCG (Merck Serono, Swiss) was injected intramuscular. After 36 h, HFF was collected during trans-vaginal ultrasound guided aspiration of oocytes. The resultant HFF samples were macroscopically clear and without contamination of the flushing medium.

The samples were centrifuged at 10,000×g at 4 °C for 30 min to produce cell debris-free HFF fraction for further analysis. Concentration of HFF was determined by the Bradford method [20]. This work has been approved by the Ethics Committee of Beijing BaoDao Obstetrics and Gynecology Hospital, and written informed consents were obtained from all participants.

### First dimension high pH RP chromatography

Equal amounts (50μg) of HFF proteins from each sample were pooled for separation. The samples were sequentially treated with 20 mM dithiothreitol at 37 °C for 120 min, and 50 mM iodoacetamide in dark for 60 min at room temperature. Then the sample was finally digested using trypsin (sequencing grade, Promega, France) (*W*/W, 1:50 enzyme/protein) overnight at 37 °C. According to the previous method with appropriate modification [21], the first dimension RP separation was performed on PF-2D HPLC System (Rigol) by using a Durashell RP column (5 μm, 150 Å, 250 mm × 4.6 mm i.d., Agela). Mobile phases A (2% acetonitrile, adjusted pH to 10.0 using NH_3_.H_2_0) and B (98% acetonitrile, adjusted pH to 10.0 using NH_3_.H_2_0) were used to develop a gradient. The solvent gradient was set as follows: 5% B, 5 min; 5–15% B, 15 min; 15–38% B, 15 min; 38–90% B, 1 min; 90% B, 8.5 min; 90–5% B, 0.5 min; 5% B, 10 min. The tryptic peptides were separated at an eluent flow rate of 0.8 ml/min and monitored at 214 nm. Totally, 28 eluent fractions were collected and dried by a SPD2010 SpeedVac concentrator system (Thermo, USA).

### Second dimension low pH RP chromatography coupled with MS/MS measurement

According to the previous method [22], the samples were dried under vacuum and reconstituted in 30 μl of 0.1% (*v*/v) formic acid, 2% (*v*/v) acetonitrile in water for subsequent analyses. Each fraction was separated and spotted using the Tempo™ LC-MALDI Spotting System (AB SCIEX, USA). Peptides were separated by a C18 AQ 150 × 0.2 mm column (3 μm, Michrom, USA) using a linear gradient formed by buffer A (2% acetonitrile, 0.1% formic acid) and buffer B (98% acetonitrile, 0.1% formic acid), from 5% to 35% of buffer B over 90 min at a flow rate of 0.5 μL/min. The eluted peptides were mixed with matrix solution (5 mg/mL in 70% acetonitrile, 0.1% trifluoroacetic acid) at a flow rate of 2 μL/min pushed by additional syringe pump. For each fraction, 616 spots were spotted on a 123× 81 mm LC-MALDI plate insert. Then the spots were analyzed using MALDI-TOF/TOF 5800 mass spectrometer (AB SCIEX, USA). A full-scan MS experiment (m/z range from 800 to 4000) was acquired, and then the top 40 ions were detected by MS/MS.

### Protein identification

Protein identification was performed with the ProteinPilot™ software (version 4.0.1; AB SCIEX). Each MS/MS spectrum was searched against a database (2017_03 released UniProtKB/Swiss-Prot human database, 20,183 entries) and a decoy database for FDR analysis (programmed in the software). The search parameters were as follows: trypsin enzyme; maximum allowed missed cleavages 1; Carbamidomethyl cysteine; biological modifications programmed in the algorithm. Proteins with high-confidence (FDR < 0.01) were considered as positively identified proteins.

### Bioinformatics

The gene ontology enrichment analysis of HFF proteins were performed by using online bioinformatics tools of PANTHER (Protein ANalysis THrough Evolutionary Relationships) classification system (released 11.1, 2016–10-24) (http://pantherdb.org/) [23] and DAVID (The Database for Annotation, Visualization and Integrated Discovery) bioinformatics resources 6.8 (https://david.ncifcrf.gov/) [24]. Each protein was placed in only one category, and those with no annotation and supporting information were categorized as “Unknown”. The pathway map of HFF proteins were achieved through KEGG: Kyoto Encyclopedia of Genes and Genomes (Release 81.0, 2017–01-01) (http://www.kegg.jp) [25]. The protein-protein interaction network for the HFF proteins was annotated using the STRING (search tool for recurring instances of neighbouring genes) database (released 10.0, 2016–04–16) (http://string-db.org/) [26]. The venn diagram was drawn through a online software “Calculate and draw custom Venn diagrams” (http://bioinformatics.psb.ugent.be/webtools/Venn/).

### Western blot analysis

According to the method described previously [27, 28], 50 μg HFF protein were separated by a 12% SDS-PAGE gel and then electronically transferred onto a nitrocellulose membrane. The resultant membrane was blocked with 5% (*w*/*v*) skimmed milk for 1 h at 37 °C, and then was incubated with the primary antibody (Abcam, Cambridge, USA, diluted 1:2000) at 4 °C overnight. After washing with TBST for three times, the membranes were incubated with horse-radish peroxidase-conjugated secondary antibody (diluted 1:5000, Zhong-Shan Biotechnology, Beijing, China) at room temperature for 1 h. The immunoreactive proteins was visualized by enhanced chemiluminescence detection reagents (Pierce, Rockford, IL, USA) (Additional file 1: Table S1).

## Results

Identification of high-confidence HFF proteome by dual RP-HPLC coupled with MALDI TOF/TOF mass spectrometry.

**Fig. 1:**
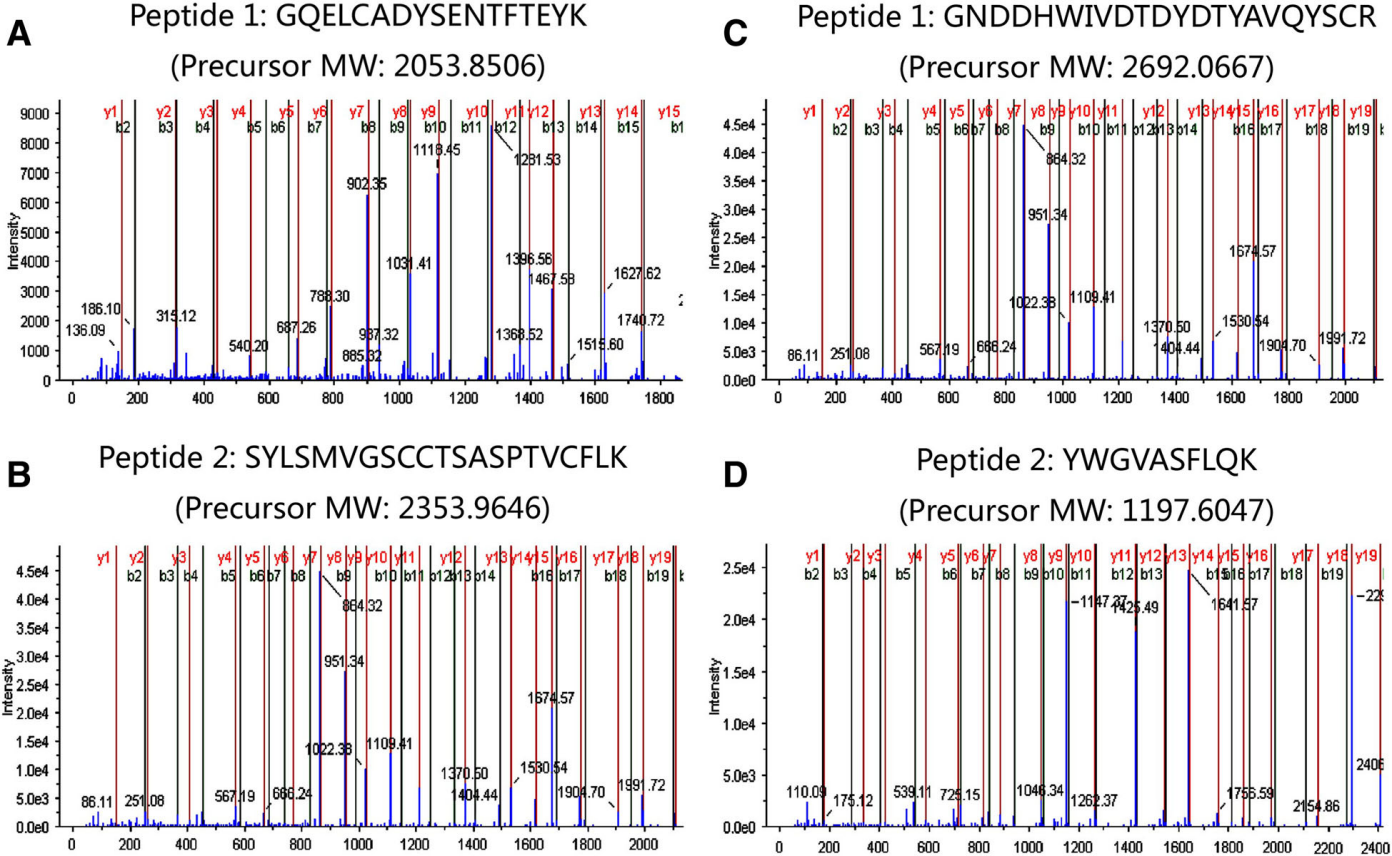
Identification of HFF proteins by LC MALDI TOF/TOF MS Spectra. The MS/MS map (**a**, **b**) marked with b ions and y ions for vitamin D-binding protein identification. The sequences of precursor at m/z 2053.8506 and 2353.9646 were analyzed by MS/MS to be GQELCADYSENTFTEYK and SYLSMVGSCCTSASPTVCFLK and the protein identified as vitamin D-binding protein. The MS/MS map (**c**, **d**) marked with b ions and y ions for retinol-binding protein 4 identification. The sequences of precursor at m/z 2692.0667 and 1197.6047 were analyzed by MS/MS to be GNDDHWIVDTDYDTYAVQYSCR and YWGVASFLQK and the protein identified as retinol-binding protein 4

**Fig. 2:**
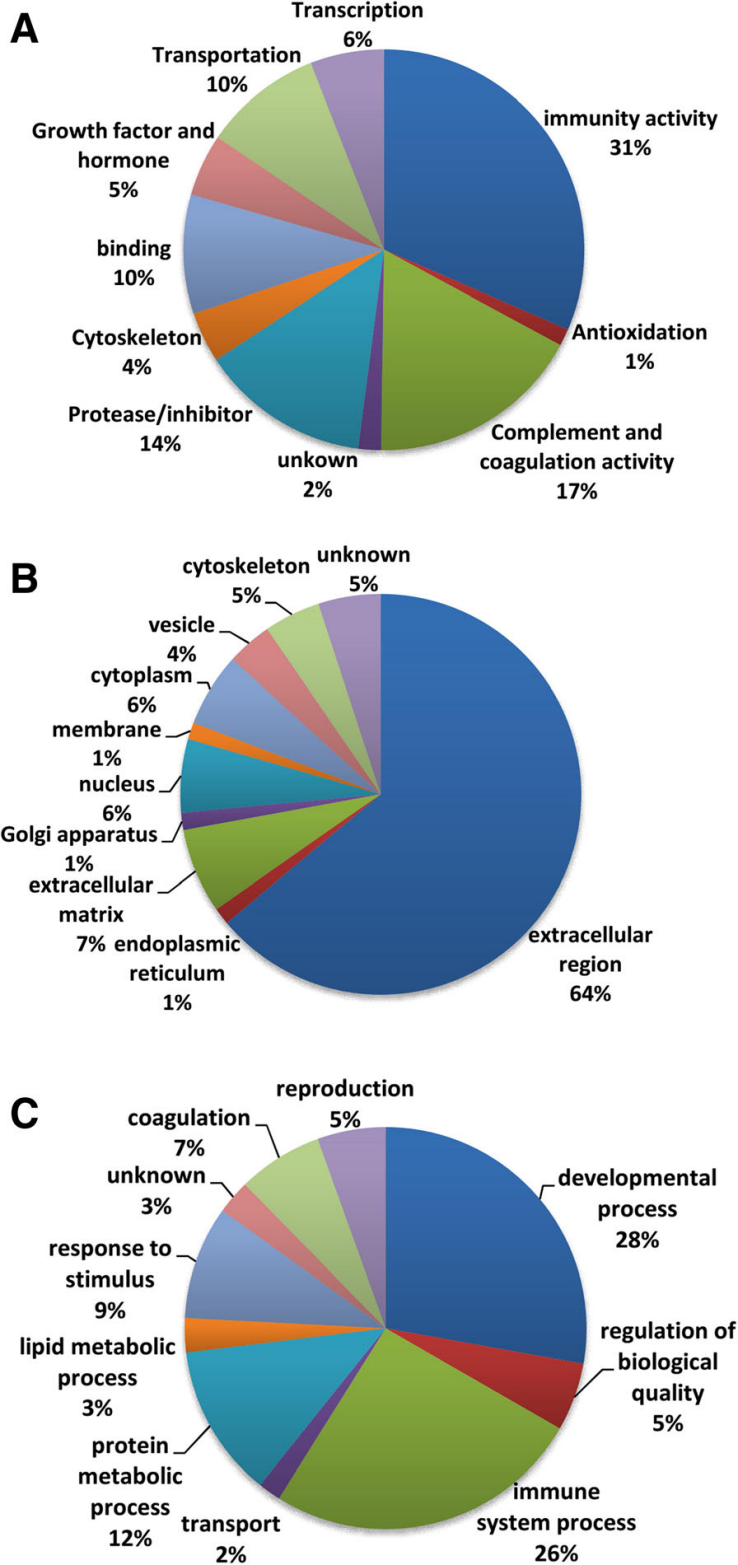
Pie diagrams of the proportion of HFF proteins categorized by GO classifications based on their (**a**) molecular function, (**b**) subcellular localization, (**c**) biological process

A peptide sequencing strategy was applied by using two-dimensional chromatography-MALDI TOF/TOF mass spectrometry. We employed high pH (pH 10) reverse phase liquid chromatography to decrease the complexity of the tryptic digest of the HFF proteins, and collected 28 fractions. Then each fraction was further separated by low pH (pH 3) reverse phase liquid chromatography, and spotted on the plate using the Tempo™ LC-MALDI Spotting System. After sequencing by a 5800 MALDI TOF/TOF mass spectrometry, the resultant spectra were analyzed by ProteinPilot™ software by searching the reviewed Swiss-Prot human database (20,183 sequences, 2017_03 released). A total of 219 unique high-confidence (FDR < 0.01) proteins were identified by two replicates (Table 1). Experiment 1 and 2 identified 188 with 2747 unique peptides and 179 proteins with 2800 unique peptides, respectively. 148 common proteins were shared between the two experiments. Figure 1 showed representative MS/MS spectra of peptides from the identified HFF proteins. The m/z of precursor (Fig. 2c) was over 2500, and almost all b-ions and y-ions were still obtained based on a 5800 MALDI TOF/TOF mass spectrometry.

**Table 1:** A list of 219 identified high-confidence HFF proteins from women underwent successful IVF by LC MALDI TOF/TOF mass spectrometry (FDR < 0.01)

No	SwissProt AC	Name protein description	Gene Name	Molecular Weight	experiment 1		experiment 2	
					Coverage(%)	Matched Peptides number	Coverage(%)	Matched Peptides number
1	P43652	Afamin	AFM	69,069	31.9	10	35.7	10
2	P02763	Alpha-1-acid glycoprotein 1	ORM1	23,512	40.8	17	40.8	15
3	P19652	Alpha-1-acid glycoprotein 2	ORM2	23,603	45.8	15	53.2	15
4	P01011	Alpha-1-antichymotrypsin	SERPINA3	47,651	53	15	44.2	16
5	P01009	Alpha-1-antitrypsin	SERPINA1	46,737	62.7	86	64.4	76
6	P04217	Alpha-1B-glycoprotein	A1BG	54,254	39.8	17	48.5	19
7	P08697	Alpha-2-antiplasmin	SERPINF2	54,566	29.1	9	47.1	11
8	P02765	Alpha-2-HS-glycoprotein	AHSG	39,325	42.8	14	55.9	18
9	P01023	Alpha-2-macroglobulin	A2M	163,291	46.8	47	47.4	46
10	P48728	Aminomethyltransferase, mitochondrial	AMT	43,946	2.2	1	-	-
11	P01019	Angiotensinogen	AGT	53,154	37.7	14	25.8	11
12	C9JTQ0	Ankyrin repeat domain-containing protein 63	ANKRD63	39,620	15	1	-	-
13	P01008	Antithrombin-III	SERPINC1	52,602	61.9	21	54.7	24
14	P02647	Apolipoprotein A-I	APOA1	30,778	73.8	67	82.4	69
15	P02652	Apolipoprotein A-II	APOA2	11,175	70	9	64	9
16	P06727	Apolipoprotein A-IV	APOA4	45,399	67.2	24	63.1	25
17	P02654	Apolipoprotein C-I	APOC1	9332	26.5	3	37.4	3
18	P02655	Apolipoprotein C-II	APOC2	11,284	39.6	2	50.5	3
19	P02656	Apolipoprotein C-III	APOC3	10,852	34.3	2	51.5	6
20	P05090	Apolipoprotein D	APOD	21,276	24.9	3	28.6	3
21	P02649	Apolipoprotein E	APOE	36,154	43.2	6	43.5	4
22	Q13790	Apolipoprotein F	APOF	35,399	-	-	8	1
23	O95445	Apolipoprotein M	APOM	21,253	26.6	2	30.3	2
24	Q9H2U1	ATP-dependent RNA helicase DHX36	DHX36	114,760	-	-	17.9	1
25	O75882	Attractin	ATRN	158,537	15	1	-	-
26	P98160	Basement membrane-specific heparan sulfate proteoglycan core protein	HSPG2	468,830	30.8	43	31	46
27	P02749	Beta-2-glycoprotein 1	APOH	38,298	51	15	41.5	16
28	Q96KN2	Beta-Ala-His dipeptidase	CNDP1	56,706	18.9	1	-	-
29	P43251	Biotinidase	BTD	61,133	9.2	2	14.2	1
30	Q7L273	BTB/POZ domain-containing protein KCTD9	KCTD9	42,567	-	-	30.1	1
31	P04003	C4b-binding protein alpha chain	C4BPA	67,033	11.9	2	27	4
32	Q96IY4	Carboxypeptidase B2	CPB2	48,424	13	2	16.1	2
33	P22792	Carboxypeptidase N subunit 2	CPN2	60,557	-	-	10.8	2
34	Q9ULM6	CCR4-NOT transcription complex subunit 6	CNOT6	63,307	-	-	2.3	1
35	Q8N8E3	Centrosomal protein of 112 kDa	CEP112	112,749	17.4	1	-	-
36	Q5SW79	Centrosomal protein of 170 kDa	CEP170	175,293	-	-	5.9	1
37	P00450	Ceruloplasmin	CP	122,205	59.6	47	58.1	58
38	O14647	Chromodomain-helicase-DNA-binding protein 2	CHD2	211,344	-	-	12	1
39	P10909	Clusterin	CLU	52,495	41.4	14	50.1	12
40	P00740	Coagulation factor IX	F9	51,778	15.2	1	-	-
41	P00742	Coagulation factor X	F10	54,732	24.6	1	14.1	1
42	P00748	Coagulation factor XII	F12	67,792	29.9	4	20.8	4
43	Q5TID7	Coiled-coil domain-containing protein 181	CCDC181	60,103	-	-	7.9	1
44	P02746	Complement C1q subcomponent subunit B	C1QB	26,722	20.2	1	18.6	1
45	Q9NZP8	Complement C1r subcomponent-like protein	C1RL	53,498	8.6	1	6.2	1
46	P06681	Complement C2	C2	83,268	21.5	4	22.7	6
47	P01024	Complement C3	C3	187,148	67.1	121	74.1	119
48	P0C0L4	Complement C4-A	C4A	192,785	46.6	53	54.8	66
49	P0C0L5	Complement C4-B	C4B	192,751	46.3	52	53	66
50	P01031	Complement C5	C5	188,305	20.3	7	27.1	12
51	P13671	Complement component C6	C6	104,786	26	6	25.5	6
52	P10643	Complement component C7	C7	93,518	35.2	8	23.1	5
53	P07357	Complement component C8 alpha chain	C8A	65,163	24.8	5	23.5	4
54	P07358	Complement component C8 beta chain	C8B	67,047	37.1	4	37.2	6
55	P07360	Complement component C8 gamma chain	C8G	22,277	48.5	7	48	5
56	P02748	Complement component C9	C9	63,173	36.5	8	35.8	10
57	P00751	Complement factor B	CFB	85,533	41.4	20	51.4	25
58	P08603	Complement factor H	CFH	139,096	55.4	43	56.9	45
59	Q03591	Complement factor H-related protein 1	CFHR1	37,651	33.9	2	27.3	5
60	P05156	Complement factor I	CFI	65,750	31.1	7	31.7	5
61	P08185	Corticosteroid-binding globulin	SERPINA6	45,141	19.5	3	17.3	2
62	Q9UBG0	C-type mannose receptor 2	MRC2	166,674	3.2	1	-	-
63	P01034	Cystatin-C	CST3	15,799	22.6	1	-	-
64	P30876	DNA-directed RNA polymerase II subunit RPB2	POLR2B	133,897	-	-	10.7	1
65	Q8NHS0	DnaJ homolog subfamily B member 8	DNAJB8	25,686	16.8	1	-	-
66	Q96DT5	Dynein heavy chain 11, axonemal	DNAH11	520,369	-	-	9.8	1
67	Q9C0C9	E2 ubiquitin-conjugating enzyme	UBE2O	141,293	-	-	3.9	1
68	O95071	E3 ubiquitin-protein ligase UBR5	UBR5	309,352	7.6	1	-	-
69	A4FU69	EF-hand calcium-binding domain-containing protein 5	EFCAB5	173,404	8.1	1	-	-
70	Q16610	Extracellular matrix protein 1	ECM1	60,674	20.7	2	11.5	2
71	Q9UGM5	Fetuin-B	FETUB	42,055	12.8	1	18.3	1
72	P02671	Fibrinogen alpha chain	FGA	94,973	44.8	40	47.6	44
73	P02675	Fibrinogen beta chain	FGB	55,928	72.1	53	68.6	42
74	P02679	Fibrinogen gamma chain	FGG	51,512	69.1	36	68	34
75	P02751	Fibronectin	FN1	262,625	30.3	33	31.2	34
76	Q08380	Galectin-3-binding protein	LGALS3BP	65,331	22.9	1	28.7	4
77	P06396	Gelsolin	GSN	85,698	43.9	16	43.6	20
78	P07093	Glia-derived nexin	SERPINE2	44,002	34.7	4	28.6	3
79	P22352	Glutathione peroxidase 3	GPX3	25,552	16.4	2	27	1
80	Q7Z4J2	Glycosyltransferase 6 domain-containing protein 1	GLT6D1	36,274	2.6	1	-	-
81	P0CG08	Golgi pH regulator B	GPR89B	52,917	-	-	7.7	1
82	P00738	Haptoglobin	HP	45,205	61.1	26	58.6	23
83	P00739	Haptoglobin-related protein	HPR	39,030	44.3	10	-	-
84	Q9Y6N9	Harmonin	USH1C	62,211	7.8	1	-	-
85	P69905	Hemoglobin subunit alpha	HBA1/HBA2	15,258	-	-	28.2	1
86	P68871	Hemoglobin subunit beta	HBB	15,998	43.5	2	52.4	1
87	P02790	Hemopexin	HPX	51,676	55.8	44	76.4	50
88	P05546	Heparin cofactor 2	SERPIND1	57,071	21	6	34.9	6
89	Q04756	Hepatocyte growth factor activator	HGFAC	70,682	5.3	1	-	-
90	P04196	Histidine-rich glycoprotein	HRG	59,578	33	15	37.9	18
91	O43365	Homeobox protein Hox-A3	HOXA3	46,369	6.5	1	-	-
92	P78426	Homeobox protein Nkx-6.1	NKX6–1	37,849	16.4	1	-	-
93	Q14520	Hyaluronan-binding protein 2	HABP2	62,672	15.4	2	11.8	3
94	P0DOX2	Immunoglobulin alpha-2 heavy chain	N/A	48,935	39.1	14	40.9	12
95	P0DOX3	Immunoglobulin delta heavy chain	N/A	56,224	19.9	1	23.4	1
96	P0DOX4	Immunoglobulin epsilon heavy chain	N/A	60,323	8.4	2	15.7	2
97	P0DOX5	Immunoglobulin gamma-1 heavy chain	N/A	49,330	70.6	144	71.9	123
98	P01876	Immunoglobulin heavy constant alpha 1	IGHA1	37,655	42.8	23	48.2	16
99	P01859	Immunoglobulin heavy constant gamma 2	IGHG2	35,901	74.9	104	69.9	92
100	P01860	Immunoglobulin heavy constant gamma 3	IGHG3	41,287	72.4	69	78.3	65
101	P01861	Immunoglobulin heavy constant gamma 4	IGHG4	35,941	79.8	101	68.8	85
102	P01871	Immunoglobulin heavy constant mu	IGHM	49,440	33.1	10	34.7	12
103	A0A0C4DH31	Immunoglobulin heavy variable 1–18	IGHV1–18	12,820	53	7	48.7	9
104	P23083	Immunoglobulin heavy variable 1–2	IGHV1–2	13,085	47.9	6	-	-
105	A0A0C4DH33	Immunoglobulin heavy variable 1–24	IGHV1–24	12,824	38.5	2	38.5	3
106	A0A0C4DH29	Immunoglobulin heavy variable 1–3	IGHV1–3	13,008	38.5	3	-	-
107	A0A0A0MS14	Immunoglobulin heavy variable 1–45	IGHV1–45	13,508	9.4	2	-	-
108	P01743	Immunoglobulin heavy variable 1–46	IGHV1–46	12,933	-	-	32.5	5
109	P01742	Immunoglobulin heavy variable 1–69	IGHV1–69	12,659	-	-	34.2	5
110	P01762	Immunoglobulin heavy variable 3–11	IGHV3–11	12,909	38.5	10	53.9	11
111	P01766	Immunoglobulin heavy variable 3–13	IGHV3–13	12,506	60.3	6	-	-
112	A0A0B4J1V0	Immunoglobulin heavy variable 3–15	IGHV3–15	12,926	55.5	8	42.9	7
113	P01764	Immunoglobulin heavy variable 3–23	IGHV3–23	12,582	60.7	15	54.7	10
114	A0A0B4J1X8	Immunoglobulin heavy variable 3–43	IGHV3–43	13,077	-	-	34.8	6
115	A0A0A0MS15	Immunoglobulin heavy variable 3–49	IGHV3–49	13,056	47.1	3	50.4	3
116	A0A075B6Q5	Immunoglobulin heavy variable 3–64	IGHV3–64	12,891	59.3	2	18.6	1
117	A0A0C4DH42	Immunoglobulin heavy variable 3–66	IGHV3–66	12,698	61.2	14	55.2	10
118	P01780	Immunoglobulin heavy variable 3–7	IGHV3–7	12,943	76.9	14	77.8	12
119	A0A0B4J1Y9	Immunoglobulin heavy variable 3–72	IGHV3–72	13,203	55.5	9	-	-
120	A0A0B4J1V6	Immunoglobulin heavy variable 3–73	IGHV3–73	12,858	58	3	58	4
121	P01782	Immunoglobulin heavy variable 3–9	IGHV3–9	12,945	51.7	8	51.7	9
122	P06331	Immunoglobulin heavy variable 4–34	IGHV4–34	13,815	-	-	38.2	4
123	P01824	Immunoglobulin heavy variable 4–39	IGHV4–39	13,917	19.2	4	-	-
124	A0A0C4DH38	Immunoglobulin heavy variable 5–51	IGHV5–51	12,675	66.7	9	50.4	8
125	P01834	Immunoglobulin kappa constant	IGKC	11,765	88.8	50	92.5	37
126	P0DOX7	Immunoglobulin kappa light chain	N/A	23,379	61.2	52	62.6	39
127	P04430	Immunoglobulin kappa variable 1–16	IGKV1–16	12,618	-	-	34.2	2
128	A0A075B6S5	Immunoglobulin kappa variable 1–27	IGKV1–27	12,712	47	8	65	8
129	P01594	Immunoglobulin kappa variable 1–33	IGKV1–33	12,848	49.6	5	42.7	4
130	P01602	Immunoglobulin kappa variable 1–5	IGKV1–5	12,782	30.8	3	30.8	6
131	A0A0C4DH72	Immunoglobulin kappa variable 1–6	IGKV1–6	12,697	47	4	47	5
132	A0A0C4DH69	Immunoglobulin kappa variable 1–9	IGKV1–9	12,715	74.4	5	44.4	5
133	P01611	Immunoglobulin kappa variable 1D-12	IGKV1D-12	12,620	44.4	5	49.6	7
134	A0A0B4J2D9	Immunoglobulin kappa variable 1D-13	IGKV1D-13	12,569	13.7	1	-	-
135	A0A075B6S4	Immunoglobulin kappa variable 1D-17	IGKV1D-17	12,835	28.2	1	43.6	2
136	P04432	Immunoglobulin kappa variable 1D-39	IGKV1D-39	12,737	47	6	47.9	6
137	P06310	Immunoglobulin kappa variable 2–30	IGKV2–30	13,185	50	5	63.3	7
138	P01615	Immunoglobulin kappa variable 2D-28	IGKV2D-28	12,957	33.3	5	40.8	5
139	A0A075B6S2	Immunoglobulin kappa variable 2D-29	IGKV2D-29	13,143	-	-	20.8	5
140	P01614	Immunoglobulin kappa variable 2D-40	IGKV2D-40	13,310	37.2	6	37.2	5
141	P04433	Immunoglobulin kappa variable 3–11	IGKV3–11	12,575	54.8	16	49.6	10
142	P01624	Immunoglobulin kappa variable 3–15	IGKV3–15	12,496	42.6	9	50.4	8
143	P01619	Immunoglobulin kappa variable 3–20	IGKV3–20	12,557	70.7	16	70.7	14
144	A0A087WSY6	Immunoglobulin kappa variable 3D-15	IGKV3D-15	12,534	42.6	10	56.5	8
145	A0A0C4DH25	Immunoglobulin kappa variable 3D-20	IGKV3D-20	12,515	64.7	10	64.7	8
146	P06312	Immunoglobulin kappa variable 4–1	IGKV4–1	13,380	34.7	10	40.5	6
147	A0M8Q6	Immunoglobulin lambda constant 7	IGLC7	11,254	54.7	13	53.8	10
148	A0A0B4J1U3	Immunoglobulin lambda variable 1–36	IGLV1–36	12,478	13.7	1	13.7	1
149	P01703	Immunoglobulin lambda variable 1–40	IGLV1–40	12,302	21.2	2	-	-
150	P01700	Immunoglobulin lambda variable 1–47	IGLV1–47	12,284	54.7	4	39.3	3
151	P01706	Immunoglobulin lambda variable 2–11	IGLV2–11	12,644	22.7	3	-	-
152	A0A075B6K4	Immunoglobulin lambda variable 3–10	IGLV3–10	12,441	40	4	40	3
153	P01714	Immunoglobulin lambda variable 3–19	IGLV3–19	12,042	50	2	42.9	1
154	P80748	Immunoglobulin lambda variable 3–21	IGLV3–21	12,446	35.9	3	-	-
155	P01717	Immunoglobulin lambda variable 3–25	IGLV3–25	12,011	-	-	43.8	3
156	P01721	Immunoglobulin lambda variable 6–57	IGLV6–57	12,566	20.5	2	-	-
157	P0DOX8	Immunoglobulin lambda-1 light chain	N/A	22,830	44.4	23	44.4	20
158	P15814	Immunoglobulin lambda-like polypeptide 1	IGLL1	22,963	23	5	23	5
159	P35858	Insulin-like growth factor-binding protein complex acid labile subunit	IGFALS	66,035	23.1	4	27.4	6
160	P16144	Integrin beta-4	ITGB4	202,167	4.9	1	-	-
161	P19827	Inter-alpha-trypsin inhibitor heavy chain H1	ITIH1	101,389	33.6	20	33.7	25
162	P19823	Inter-alpha-trypsin inhibitor heavy chain H2	ITIH2	106,463	35.9	18	42.6	20
163	Q06033	Inter-alpha-trypsin inhibitor heavy chain H3	ITIH3	99,849	5.2	1	15.5	1
164	Q14624	Inter-alpha-trypsin inhibitor heavy chain H4	ITIH4	103,357	38.4	23	47	26
165	Q15811	Intersectin-1	ITSN1	195,422	-	-	9.9	1
166	P29622	Kallistatin	SERPINA4	48,542	26.5	4	23	5
167	Q92764	Keratin, type I cuticular Ha5	KRT35	50,361	-	-	16.7	1
168	P13645	Keratin, type I cytoskeletal 10	KRT10	58,827	5.8	1	-	-
169	P04264	Keratin, type II cytoskeletal 1	KRT1	66,039	23.6	3	30	2
170	P01042	Kininogen-1	KNG1	71,957	53.7	25	41	23
171	P02750	Leucine-rich alpha-2-glycoprotein	LRG1	38,178	21.6	4	27.1	5
172	P18428	Lipopolysaccharide-binding protein	LBP	53,384	14.8	1	13.3	1
173	P51884	Lumican	LUM	38,429	30.2	3	27.8	3
174	P14174	Macrophage migration inhibitory factor	MIF	12,476	18.3	2	-	-
175	P01033	Metalloproteinase inhibitor 1	TIMP1	23,171	18.8	2	34.8	2
176	Q7Z5P9	Mucin-19	MUC19	805,253	4.3	1	-	-
177	P35579	Myosin-9	MYH9	226,532	-	-	15.8	1
178	Q96PD5	N-acetylmuramoyl-L-alanine amidase	PGLYRP2	62,217	26	7	29.3	6
179	A6NHN0	Otolin-1	OTOL1	49,422	15.3	1	-	-
180	P04180	Phosphatidylcholine-sterol acyltransferase	LCAT	49,578	15.5	2	-	-
181	P36955	Pigment epithelium-derived factor	SERPINF1	46,312	22.3	5	17.9	5
182	P03952	Plasma kallikrein	KLKB1	71,370	23	6	26.5	6
183	P05155	Plasma protease C1 inhibitor	SERPING1	55,154	34.8	9	33.2	16
184	P05154	Plasma serine protease inhibitor	SERPINA5	45,675	13.6	3	-	-
185	P00747	Plasminogen	PLG	90,569	63	30	58.8	32
186	Q96GD3	Polycomb protein SCMH1	SCMH1	73,354	4.7	1	-	-
187	Q8WUM4	Programmed cell death 6-interacting protein	PDCD6IP	96,023	-	-	14.1	1
188	P46013	Proliferation marker protein Ki-67	MKI67	358,694	11.9	1	21.8	1
189	P15309	Prostatic acid phosphatase	ACPP	44,566	25.1	4	17.9	2
190	P02760	Protein AMBP	AMBP	38,999	38.9	11	42.1	12
191	Q9UK55	Protein Z-dependent protease inhibitor	SERPINA10	50,707	15.5	2	18.9	2
192	Q96PF1	Protein-glutamine gamma-glutamyltransferase Z	TGM7	79,941	-	-	7.5	1
193	P00734	Prothrombin	F2	70,037	59.8	33	62.4	31
194	P02753	Retinol-binding protein 4	RBP4	23,010	40.3	11	55.7	13
195	O94885	SAM and SH3 domain-containing protein 1	SASH1	136,653	-	-	10.3	1
196	P04279	Semenogelin-1	SEMG1	52,131	30.5	5	32.3	5
197	Q02383	Semenogelin-2	SEMG2	65,444	21	3	18	5
198	P57059	Serine/threonine-protein kinase SIK1	SIK1	84,902	-	-	7.3	1
199	P02787	Serotransferrin	TF	77,064	71.4	143	79.4	185
200	P02768	Serum albumin	ALB	69,367	89.3	607	91.3	550
201	P35542	Serum amyloid A-4 protein	SAA4	14,747	30	2	49.2	6
202	P02743	Serum amyloid P-component	APCS	25,387	26.5	5	25.1	5
203	P27169	Serum paraoxonase/arylesterase 1	PON1	39,731	24.5	7	19.2	5
204	P04278	Sex hormone-binding globulin	SHBG	43,779	18.7	4	21.9	3
205	P09486	SPARC	SPARC	34,632	-	-	5.3	1
206	Q6N022	Teneurin-4	TENM4	307,957	5.3	1	-	-
207	P05452	Tetranectin	CLEC3B	22,537	22.8	2	30.2	2
208	P05543	Thyroxine-binding globulin	SERPINA7	46,325	14.5	1	23.6	2
209	Q8WZ42	Titin	TTN	3,816,030	10.6	1	-	-
210	P21675	Transcription initiation factor TFIID subunit 1	TAF1	212,677	-	-	7	1
211	Q66K66	Transmembrane protein 198	TMEM198	39,475	2.5	2	2.5	1
212	P02766	Transthyretin	TTR	15,887	69.4	12	69.4	19
213	P13611	Versican core protein	VCAN	372,820	-	-	5.2	2
214	P02774	Vitamin D-binding protein	GC	52,964	63.9	29	60.3	28
215	P04070	Vitamin K-dependent protein C	PROC	52,071	-	-	2.2	1
216	P07225	Vitamin K-dependent protein S	PROS1	75,123	12.6	2	-	-
217	P04004	Vitronectin	VTN	54,306	32.6	11	32.2	15
218	Q6PF04	Zinc finger protein 613	ZNF613	70,143	6.6	1	-	-
219	P25311	Zinc-alpha-2-glycoprotein	AZGP1	34,259	52.7	14	52	17

### Bioinformatics analysis of the HFF proteome

The proteins identified by mass spectrometry were broadly placed into several GO categories on the basis of the PANTHER, DAVID and PubMed databases (Fig. 2). Based on molecular function, the majority (31%) of proteins were related to immunity, whereas other involved protein functions were mainly complement and coagulation (17%), protease or inhibitor (14%), and transportation (10%) (Fig. 2a). Based on subcellular localization, the majority (64%) of the identified proteins located in extracellular region. Other main locations were extracellular matix (7%), nuleus (6%), and cytoskeleton (5%) (Fig. 2b). Based on biological process, the majority (28%) of proteins was related to developmental process, and the next prevalence was immunological system process (26%). The other groups were involved into protein metabolic process (12%), reproduction (5%), lipid metabolic process (3%), and transportation (2%) (Fig. 2c).

**Fig. 3:**
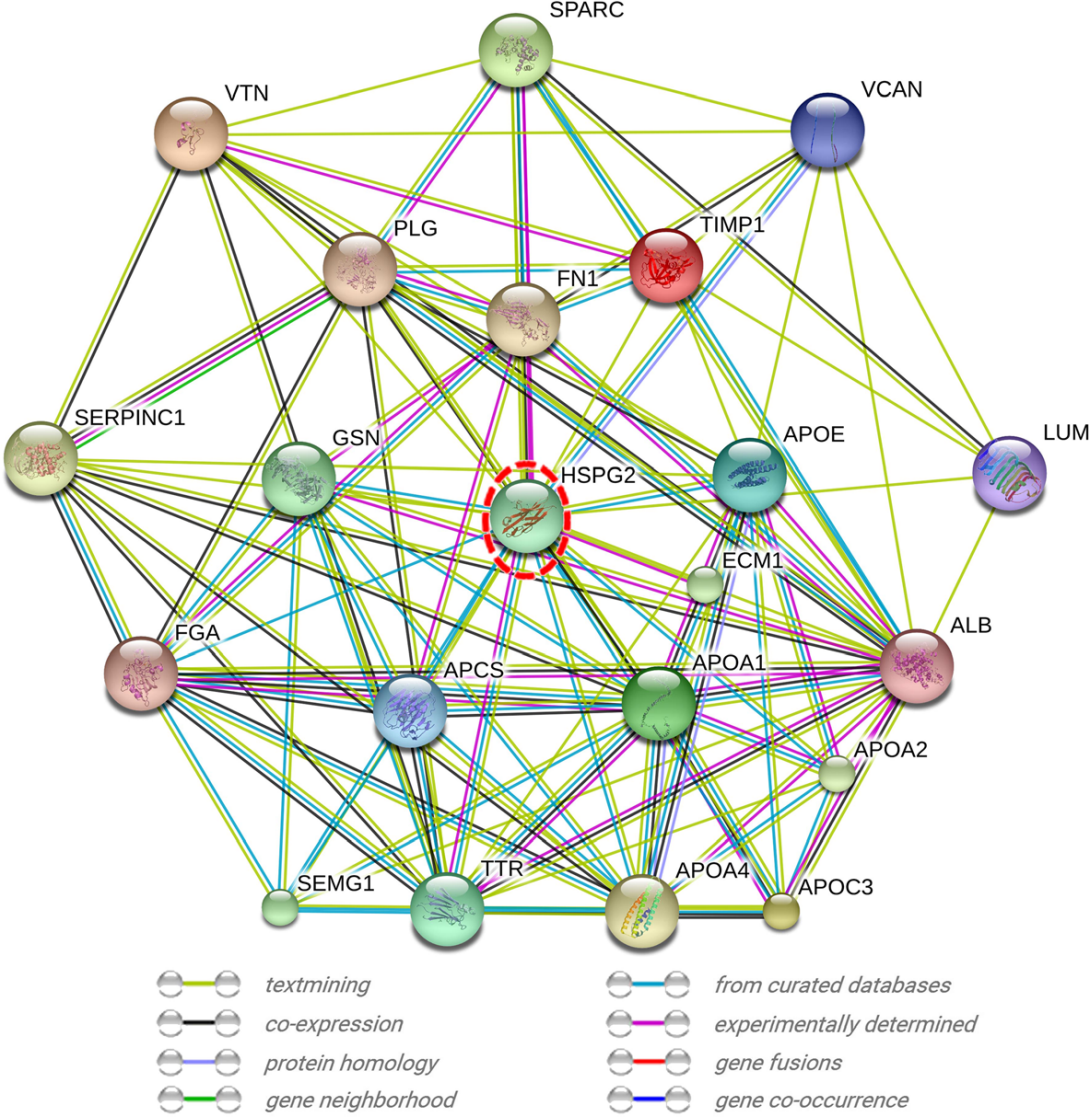
Presentative Network of protein HSPG2 in the identified HFF proteome. A total of 21 genes are connected with 105 paired relationships annotated by STRING database. The relationships among proteins were derived from evidence that includes textmining, co-expression, protein homology, gene neighborhood, from curated databases, experimentally determined, gene fusions, and gene co-occurrence (as shown in the legend with different color)

KEGG pathway analysis was performed to map HFF protein interactions, Pathways associated with complement and coagulation cascades (P_Value = 5.8E-52), vitamin digestion and absorption (P_Value = 0.023), and (P_Value = 0.066) were significantly enriched. Figure 3 showed the complement and coagulation cascades pathway which included 17 (7.8%) and 21 (9.6%) highlighted HFF proteins in coagulation cascade and complement cascade, respectively.

**Fig. 4:**
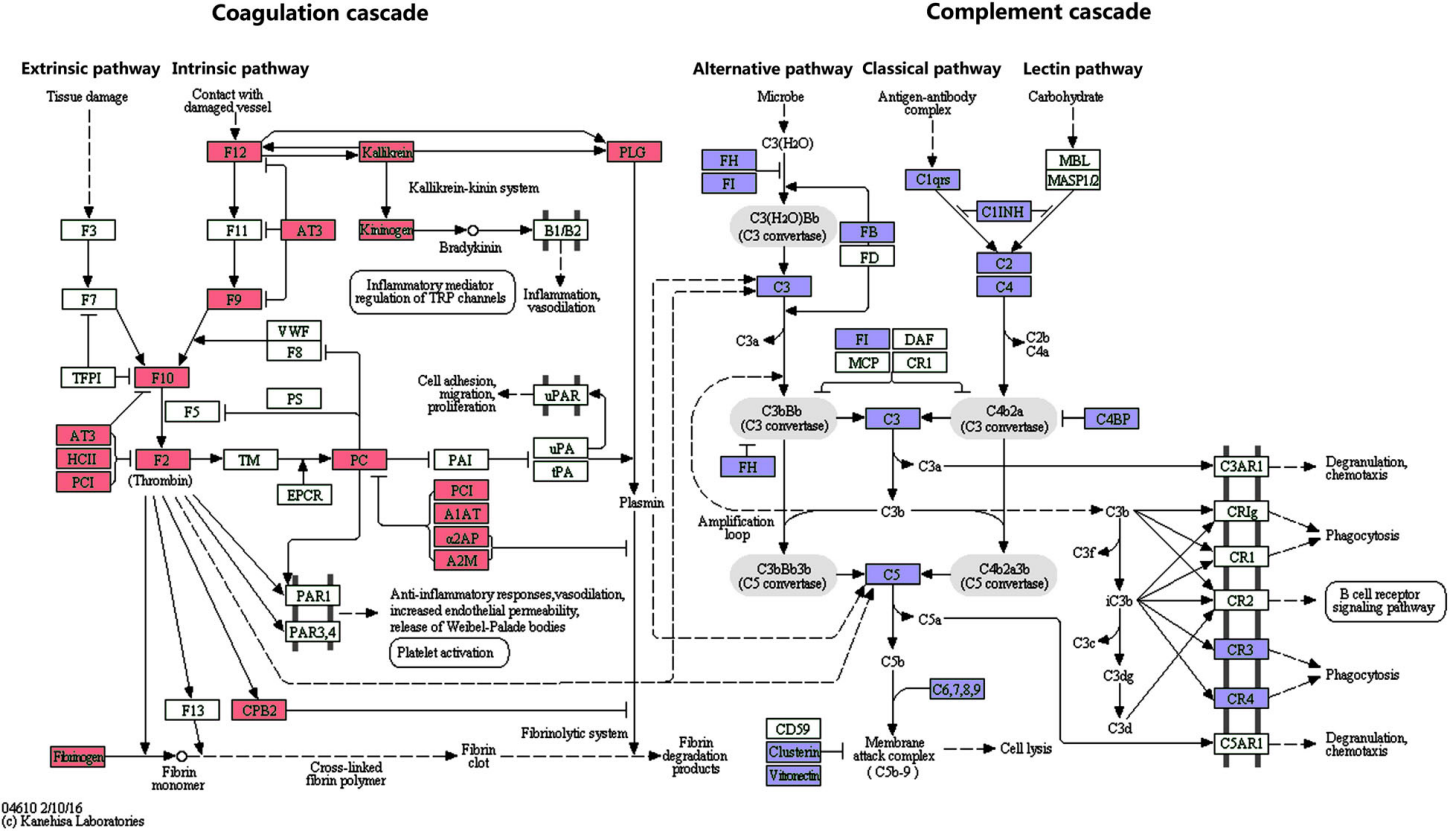
The KEGG pathway of complement and coagulation cascades with the identified HFF proteins highlighted. Generated by the KEGG online (hsa04610), this diagram showed the roles if HFF proteins in complement (*Red*) and coagulation cascades (*Blue*)

A protein-protein interaction network was constructed by retrieving the STRING database. 151 proteins were in connection with other proteins, which lead to 738 paired relationships. As an example, 21 of 151 proteins related to basement membrane-specific heparan sulfate proteoglycan core protein (HSPG) was chosen, and 105 paired relationships were connected (Fig. 4).

### Comparison of present HFF proteome, the previous reported HFF proteome and human plasma proteome

**Fig. 5:**
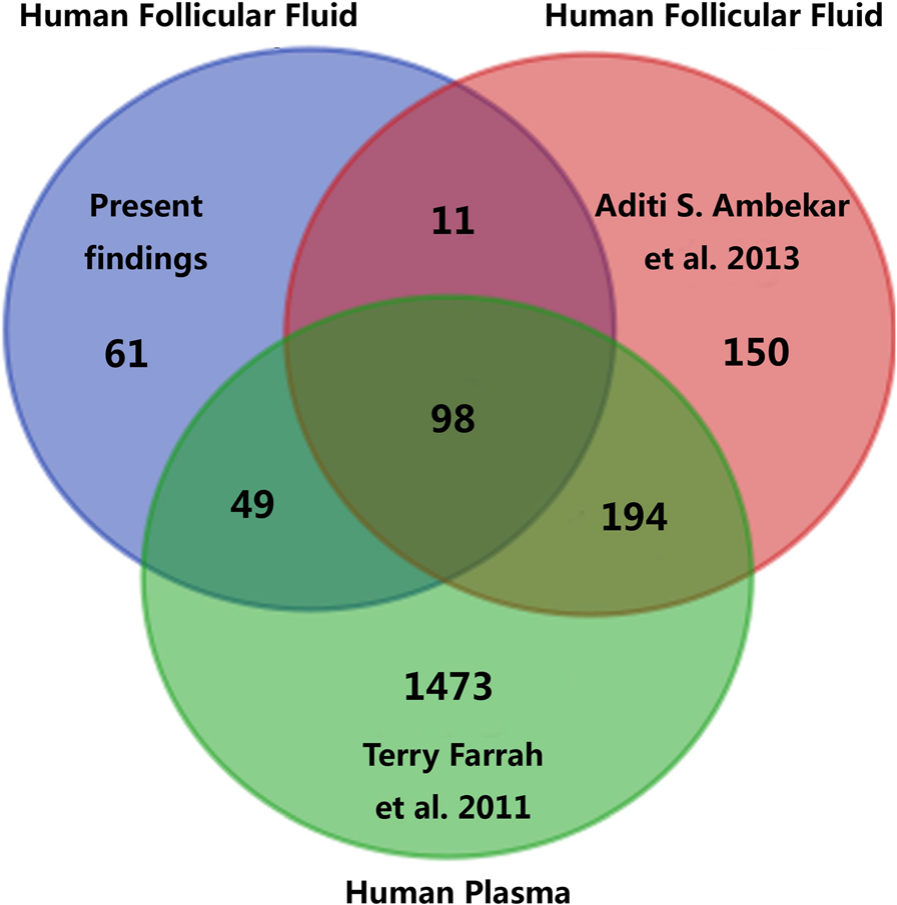
Venn diagram of the overlap of HFF and human plasma protein datasets. Distribution of our present findings or the previously reported HFF proteins (Aditi S. Ambekar et al. 2013) and their overlap with those reported in human plasma (Terry Farrah et al. 2011)

To disclose the overlap of the HFF proteomes between different labs and to explore the orign of the HFF proteins, the previous reported HFF proteins [14] and the human plasma proteome [29] were selected, whose protein identification criteria were both at a false discovery rate (FDR) of 1%. The results reflected the overlap of our HFF proteins and the previously reported HFF proteins with human plasma proteins (Fig. 5). A total of 49% proteins in our HFF data were common to the previous HFF data. Compared with human plasma proteins, 69% proteins from our HFF data and 64% proteins from previous HFF data were common to human plasma proteins.

### Western blotting analysis

**Fig. 6:**
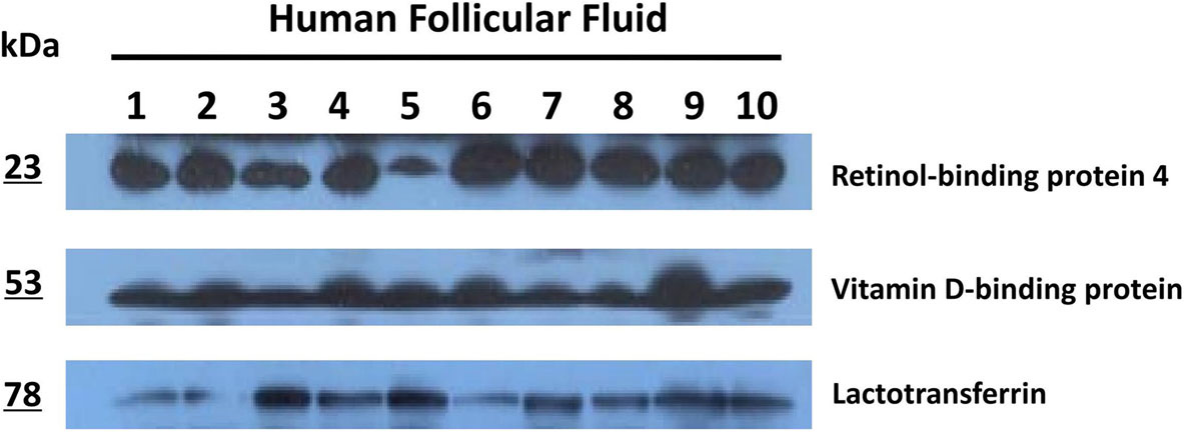
Immunoblot analysis of retinol-binding protein 4, vitamin D-binding protein and lactotransferrin in 10 HFF samples of women underwent successful IVF. Protein lysates prepared from 10 HFF samples were examined by immunoblots using specific antibodies recognizing the retinol-binding protein 4(23 kDa), vitamin D-binding protein (53 kDa) and lactotransferrin (78 kDa)

To verify the confidence of the proteome data, the expression patterns of 3 HFF proteins (retinol-binding protein 4, vitamin D-binding protein and lactotransferrin) from 10 women undergoing successful IVF were analyzed by western blotting (Fig. 6). Those three proteins could be detected in all 10 HFF samples. Compared with retinol-binding protein 4 and lactotransferrin, the expression of vitamin D-binding protein was relatively constant level in the HFF of ten women.

## Discussion

Proteomics has been carried out to discover HFF biomarkers for decades, and liquid chromatography coupled with ion trap MS became widely available with the development of high-throughput sequencing. The identification of HFF proteins from women with and without endometriosis was performed using ESI MS/MS [30]. Nanoflow LC-MS/MS combined with TMT labeling was used to identify HFF biomarkers from women undergoing IVF/ICSI treatment with or without folic acid supplement [31]. Another advance LTQ Orbitrap system coupled with LC was also applied to comparing HFF proteins between fertilized oocytes and non-fertilized oocytes from the same patient [32]. Based on sample pre-fractionation using microscale in-solution isoelectric focusing (IEF), capillary electrophoresis (CE) coupled off-line to matrix assisted laser desorption/ionization time of flight tandem mass spectrometry (MALDI TOF MS/MS) identified 73 unique proteins [33]. Hanrieder and co-workers [34] utilized a proteomic strategy of IEF and reversed-phase nano-liquid chromatography coupled to MALDI TOF/TOF mass spectrometry to identify 69 proteins related to controlled ovarian hyper stimulation (COH) during IVF. However, limited proteins were identified which delayed the research of HFF protein networks.

In the present work, a dual RP-HPLC coupled with MALDI TOF/TOF mass spectrometry was performed to identify HFF protein profiles associated with successful IVF, and 219 unique high-confidence (FDR < 0.01) HFF proteins were identified by searching the reviewed Swiss-Prot human database (20,183 sequences). Meanwhile, the new strategy indicated that the effective dual reverse LC pre-fractionation [21] could identify more HFF proteins.

Ambekar and co-workers carried out SDS-PAGE, OFFGEL and SCX-based separation followed by LC–MS/MS (an LTQ-Orbitrap Velos MS) to identify 480 HFF proteins with high confidence (FDR < 0.01) [14]. A comparison with our results and these data showed that more than 50% proteins in present study were not found in previous dataset (Additional file 2: Figure S1), which indicated that the data from different MS platforms were complementary. Retinol-binding protein 4 and vitamin D-binding protein were verified by western blotting, and the results showed they were all expressed in the 10 HFF samples. Lactotransferrin was uniquely included in Ambekar’s data, and was also successfully detected by western blotting in our study. This result not only testified the good quality of Ambekar’s data, but also facilitated to integrate the data from different MS platform in the future. Interestingly, more than 60% of combined HFF proteins from our data and Ambekar’s data were found in the reported human plasma data [29]. HFF was a complex mixture, and the content of HFF mainly originates from the transfer of blood plasma constituents via theca capillaries, and the secretion of granulosa and thecal cells [5]. From the above contrast, we considered the transfer of plasma proteins was the major source of HFF, and the alternative permeability of theca capillaries would change the HFF compositions which inevitably impaired the oocyte quality, and even caused unsuccessful IVF outcome.

Bioinformatics analysis showed that 5% HFF proteins were involved in lipid metabolism and transport process. It has been reported that ageing could decrease apolipoprotein A1 and apolipoprotein CII, while increase apolipoprotein E, which were associated with the decline in production of mature oocytes and the decline in fertility potential [35]. Preconception folic acid supplementation upregulated apolipoprotein A-I and apolipoprotein C-I of the HDL pathway in human follicular fluid, which increased embryo quality and IVF/ICSI treatment outcome [30]. In our HFF data, apolipoprotein A-I, apolipoprotein A-II, apolipoprotein A-IV, apolipoprotein C-I, apolipoprotein C-II, apolipoprotein C-III, apolipoprotein D, apolipoprotein E, apolipoprotein F, and apolipoprotein M were all found, which indicated that those apolipoproteins were related to cholesterol homeostasis and steroidogenesis and played important roles in the maintenance of oocyte maturation microenvironment.

Pathway analysis showed that complement and coagulation cascades were the most prominent pathways (P_Value = 5.8E-52). Complement cascade promoted coagulation through the inhibition of fibrinolysis, and coagulation cascade in return amplified complement activation. Complement cross_talked with coagulation in a reciprocal way [36]. For example, plasmin, thrombin, elastase and plasma kallikrein could activate C3 [37]. Coagulation activation factor XII could cleave C1 to activate the classical complement pathway [38]. And thrombin could also directly cleave C5 to generate active C5a [39]. Among our HFF proteins, components (F12, KLKB1, PLG, KNG1, F9, F10, SERPINC1, SERPIND1, SERPINA5, F2, PROS1, PROC, SERPINA1, SERPINF2, A2M, CPB2, and FGA) of extrinsic pathway and intrinsic pathway in coagulation cascade and those (FH, FI, FB, C3, C1qrs, SERPING1, C2, C4, C4BP, C5, C6, C7, C8A, C8B, C8G, C9, FGA, FGG, PLG, FGB, F10) of alternative pathway, classical pathway, and lectin pathway in complement cascade were all identified. During follicle development and ovulation, coagulation system in HFF contributed to HFF liquefaction, fibrinolysis and the breakdown of follicle wall [40, 41]. Follicle development had been hypothesized as the controlled inflammatory processes in 1994 [42], and inappropriate complement activation was linked to abortion [43]. Inhibition of complement activation improved angiogenesis failure and rescued pregnancies [44]. The paired comparison of HFF with plasma showed C3, C4, C4a, and C9 as well as complement factor H and clusterin might contribute to the inhibition of complement cascade activity for women undergoing controlled ovarian stimulation for IVF [45]. However there were still debates on the role of complement cascade in IVF. Physiologic complement activation protected the host against infection in normal pregnancy [46]. In comparison with those non-fertilized oocytes, C3 was more abundant in HFF from fertilized oocytes [47]. In the course of IVF treatment, the functions of complement and coagulation cascade were very complicated during ovarian hyperstimulation. More works were still deserved in both mechanism research and clinical practice.

Based on the analysis of STRING, we discovered a profound HFF protein-protein interaction networks. 151 of 219 HFF proteins participated in the network with 738 paired relationships. Basement membrane-specific HSPG was found as a node, which was also a potential biomarker for oocyte maturation in HFF. HSPG was widely distributed on the surface of animal cells, and especially strongly expressed in granulosa cells. HSPG played a critical role in controlling inflammation control through binding and activating antithrombin III during folliculogenesis [48]. Women with PCOS showed HSPG defect in follicular development [49], and on the contrary, HSPG was up-regulated in the fertilized-oocyte HFF [32]. In the network, HSPG interacted with 20 of 219 HFF proteins, and constructed 105 paired relationships. We deduced that the loss of HSPG might affect the function of the whole network or more complicated interaction maps, which might cause subsequent failures of oocyte maturation, fertilization, and IVF treatment.

## Conclusions

HFF had a natural advantage for the noninvasive prediction of oocyte quality and IVF treatment outcome. The present study would provide a new complementary dataset for better understanding of oocyte maturation, and also delineate a new networks and pathways involved into the folliculogenesis. Furthermore, those novel findings would facilitate to testify the potential biomarkers associated with oocyte quality and IVF outcome. In the future, international laboratory collaboration should be established to standardize and optimize experimental design, patient selection, HFF handling, analysis methods, data standard, and clinical verification, which will greatly promote basic research of reproductive medicine, and ultimately accelerate the clinical transformation.

## Additional files

Additional file 1: The information of antibodies and secondaries for Western blotting. (XLSX 10 kb)

Additional file 2: The overlap of known data and novel findings. (JPEG 1344 kb)

## Abbreviations

2D–GE: Two-dimensional gel electrophoresis
A2M: Alpha-2-macroglobulin
BMI: Body mass index
C1qrs: Complement C1q A chain
C2: Complement C2
C3: Complement C3
C4: Complement C4
C4BP: C4b-binding protein alpha chain
C5: Complement C5
C6: Complement C6
C7: Complement C7
C8A: Complement component C8 alpha chain
C8B: Complement component C8 beta chain
C8G: Complement component C8 gamma chain
C9: Complement C9
CE: Capillary electrophoresis
COH: Controlled ovarian hyperstimulation
CPB2: Carboxypeptidase B2
DAVID: The database for annotation, visualization and integrated discovery
F10: Coagulation factor X
F12: Coagulation factor XII
F2: Prothrombin
F9: Coagulation factor IX
FB: Complement factor B
FDR: False Discovery Rate
FDR: False discovery rate
FGA: Fibrinogen alpha chain
FGB: Fibrinogen beta chain
FGG: Fibrinogen gamma chain
FH: Complement factor H
FI: Complement factor I
HCG: Human chorionic gonadotrophin
HFF: Human follicular fluid
HSPG: Heparan sulfate proteoglycan core protein
IEF: Isoelectric focusing
IVF: In vitro fertilization
KEGG: Kyoto encyclopedia of genes and genomes
KLKB1: Plasma kallikrein
KNG1: Kininogen-1
MALDI TOF/TOF: Matrix-assisted laser desorption/ionization time-of-flight tandem
PANTHER: Protein analysis through evolutionary relationships
PCOS: Polycystic ovary syndrome
PLG: Plasminogen
PROC: Vitamin K-dependent protein C
PROS1: Vitamin K-dependent protein S
RP-HPLC: Reverse phase high performance liquid chromatography
SCX: Strong cation exchange
SDS-PAGE: One dimensional sodium dodecyl polyacrylamide gel electrophoresis
SELDI-TOF-MS: surface-enhanced laser desorption/ionization-time of flight-mass spectrometry
SERPINA1: Alpha-1-antitrypsin
SERPINA5: Plasma serine protease inhibitor
SERPINC1: Antithrombin-III
SERPIND1: Heparin cofactor 2
SERPINF2: Alpha-2-antiplasmin
SERPING1: Plasma protease C1 inhibitor
STRING: search tool for recurring instances of neighbouring genes
WCX: weak cation-exchange
WGOC: Working Group on Obesity in China
