# Supplementary figures and images for: Proteomic analysis of human follicular fluid associated with successful in vitro fertilization
Source: Reprod Biol Endocrinol. 2017 Jul 27;15:58. doi: 10.1186/s12958-017-0277-y (PMC5530927; doi:10.1186/s12958-017-0277-y)

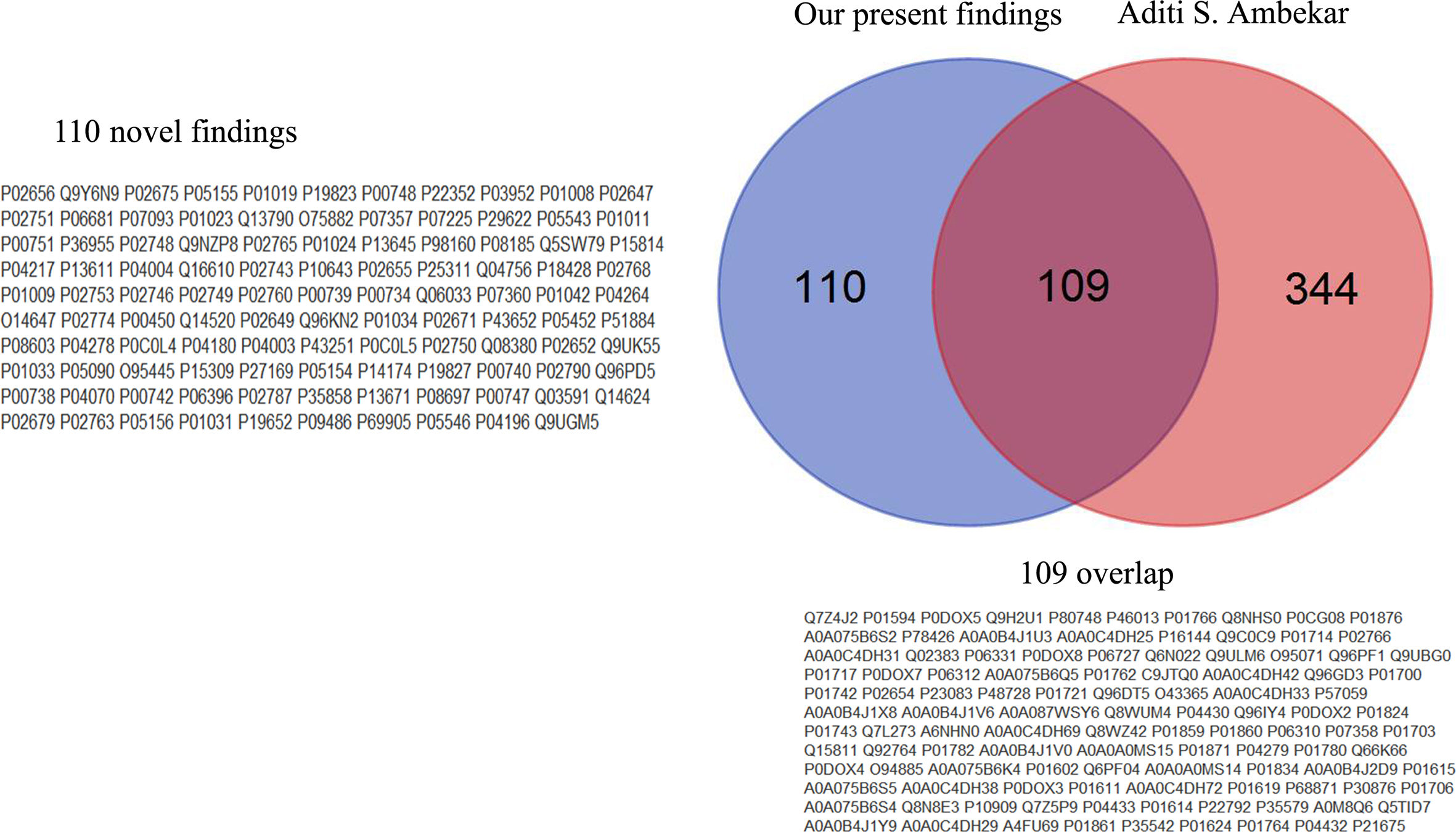

Supplement: Supplementary file 2 — The overlap of known data and novel findings. (JPEG 1344 kb) [file 12958_2017_277_MOESM2_ESM.jpg]
